# DCG++: A data-driven metric for geometric pattern recognition

**DOI:** 10.1371/journal.pone.0217838

**Published:** 2019-06-06

**Authors:** Jiahui Guan, Fushing Hsieh, Patrice Koehl

**Affiliations:** 1 Department of Statistics, University of California Davis, Davis, CA, United States of America; 2 Department of Computer Science and Genome Center, University of California Davis, Davis, CA, United States of America; University of Ulm, GERMANY

## Abstract

Clustering large and complex data sets whose partitions may adopt arbitrary shapes remains a difficult challenge. Part of this challenge comes from the difficulty in defining a similarity measure between the data points that captures the underlying geometry of those data points. In this paper, we propose an algorithm, DCG++ that generates such a similarity measure that is data-driven and ultrametric. DCG++ uses Markov Chain Random Walks to capture the intrinsic geometry of data, scans possible scales, and combines all this information using a simple procedure that is shown to generate an ultrametric. We validate the effectiveness of this similarity measure within the context of clustering on synthetic data with complex geometry, on a real-world data set containing segmented audio records of frog calls described by mel-frequency cepstral coefficients, as well as on an image segmentation problem. The experimental results show a significant improvement on performance with the DCG-based ultrametric compared to using an empirical distance measure.

## Introduction

Given a set of objects O, usually referred to as data points, each characterized by some measured properties, or features D, it is natural to think of comparing them and possibly grouping them into categories, such that objects that belong to the same category are deemed to be more similar to each other than to objects in other categories. In this context, similarity is defined by comparison of the features. This way of organizing data is the underlying mechanism behind categorization, a fundamental process currently used in nearly all scientific endeavors. The choice of similarity measure, or equivalently of a distance between objects built from their features is still an unsolved problem, usually referred to as the metric learning problem [1–3]. In this paper, we focus on this concept of distance between data points, and how the choice of such a distance influences the quality of classification of the data points, as measured by clustering applications. We emphasize that the idea of distance and its properties are not universal and depend on the domain of application. In physics for example, there are usually well established theories behind the data points that are studied; those theories define the metric to be used when comparing those data points. However, in biological problems notions of distance are usually defined from some intuitively attractive measures of similarity; it is unclear as to how much significance can be attached to such distances that may not be metric, particularly at large scales. In addition, the data themselves may be embedded into a complex manifold that cannot be detected by linear procedures. The classical K-means clustering procedure for example assumes compact, convex clusters, such that data points within a cluster are nearer to each other than they are to data points in other clusters; this may not be true if the data are embedded in non convex clusters. Multiple solutions have been developed to solve this problem. Most rely on the idea of defining a local metric that captures the geometry of the data. Examples include the definition of a geodesic distance for dimension reduction, the ISOMAP procedure [4], the introduction of a Gaussian kernel to capture local neighborhoods around data points as implemented in spectral clustering techniques [5], in diffusion maps methods [6, 7], or for defining density peaks [8], or more sophisticated topological and geometric approaches to capture the hierarchical organization of the data [9–12].

Most of the methods that implement a concept of a local metric rely on the construction of an *ϵ*-graph on the data, where *ϵ* is a parameter that defines the size of the neighborhood of a data point. This parameter is either set to a bright cutoff, such as in the original implementation of ISOMAP [4], or to the width *a* of a Gaussian kernel, as it is usually implemented in spectral clustering techniques [13]. The values given to *ϵ* is clearly data dependent, and usually set by trials and errors. Following previously published preliminary studies [14, 15] we argue in this paper that exploring the range of possible values for the scale parameter *ϵ* allows us to automatically capture the hierarchical geometry of the data points under study, much akin to the persistent homology used in topological data analysis [10]. Based on this idea, we proposed a method inspired from statistical physics that makes use of a temperature parameter *T* (equivalent to the *ϵ* parameter) to monitor phase transitions [14]. Similar to the graph theoretic approaches, we assimilated the set of data points to a weighted graph, with the weight of an edge set to a function of the empirical distance between the corresponding vertices, and the temperature factor *T*. By equating this weight with a ferromagnetic potential, the weighted graph is seen as equivalent to a potential landscape, typically characterized by many wells with various depths. It is then possible to explore this landscape and therefore define its geometry by using a dynamic Monte Carlo approach. A random walk identifies the many wells of the potential, as well as the probability of jumping from one well to another. This leads to a new weighted graph on the data, whose weights are temperature dependent. Similar to spectral clustering, we then study the Laplacian of that graph. Analysis of the eigenvectors and eigenvalues provides information about the number of clusters and corresponding cluster membership of the data points. By repeating this procedure at different temperatures, we derived the geometric hierarchy of the data points in the form of an ultrametric matrix than can then be used as input to traditional clustering techniques [15]. This method is similar in spirit to the granular model, which achieves clustering by a sequence of phase transitions on a paramagnetic potential landscape [16, 17].

This paper develops previous preliminary studies [14, 15]. In those studies, we had introduced the concept of computing an ultrametric matrix over a set of data points using the method described above, and dubbed DCG, for Data Cloud Geometry. Implementations of this method, however, were of limited use because of high computing costs and the need to significant manual tuning. In this paper we describe a complete rewriting of the algorithm that implement this methods, with the two main goals of reducing its computational cost and improving its automation. In particular, we have developed a procedure for automatic detection of the temperatures leading to phase transitions for the ferromagnetic potential. We have implemented a spectral clustering algorithm for analyzing the weighted probability graph generated with the Monte Carlo random walks, with an automatic detection of clusters. Finally, we have fully rewritten its implementation in a new software package, DCG++, written in C++ with modest parallelization such that it can be used on moderate size data sets, with up to tens of thousand of data points.

The rest of this paper is organized as follows. The next section covers related work. In Section 3, we describe our algorithm and its implementation. Section 4 presents and discusses the results obtained by our algorithm on synthetic as well as real test cases. We conclude the paper with a discussion on future developments of the method itself.

## Related work

Our focus in this paper is metric learning, namely the derivation of a metric (in fact even an ultrametric) from the data directly. The method we have developed for this problem (this work, and two preliminary studies, see [14, 15]) is intricately related to clustering, as it is by monitoring how data points cluster at multiple scales that we design our distance measure. We review here some of the clustering techniques, namely those that are derived from physics, the graph theoretic methods, and the diffusion maps, that are most related to those used in our procedure.

### Statistical physics and clustering

The idea of adapting a technique from statistical physics to perform clustering of data points is not new [18]. Following the observation that clusters appear naturally in Potts-like models, Domany and colleagues [16, 17], based on ideas related to the Ising model [19, 20], the Potts model [21], and their generalization in the random cluster model [22], proposed that the clustering problem can be formulated as the relaxation of a ferromagnetic Potts-like model. The relaxation terminates at some minimum of an energy function, and points with the same spin value are then assigned to the same cluster. The energy function is akin to the Hamiltonian of a Potts model,

$$H=\sum_{\left(i,j\right)}J_{ij}\delta \left(s_{i},s_{j}\right)$$ (1)

where *s*_*i*_ is the (integer) state of data point *i*, *δ* is the Dirac delta function (namely *δ*(*a*, *b*) = 1 if *a* = *b*, and 0 otherwise), and *J*_*ij*_ is a positive decreasing function of the distance between the two points *i* and *j*. In their original formulation, Domany and colleagues have set *J* to be a Gaussian, i.e. $J\left(i,j\right)=\text{exp}(-\frac{d{\left(i,j\right)}^{2}}{2a^{2}})$ where *d*(*i*, *j*) is the given distance between points *i* and *j*, and a width *a* that relates to the “scale” of the data, set to the average nearest neighbor distance among all pairs of points [16]. The choice of the scale however is problem dependent and somewhat arbitrary [6, 8, 16]. In addition to the applications of statistical physics techniques, it is worth mentioning the use of quantum mechanics for clustering [23], leading to the concept of quantum clustering [24, 25].

### Graph theoretic algorithms for clustering

The idea of expressing clustering as a graph partitioning problem has been explored in many different forms [26–32]. All those methods use a graph representation of the data. Formally, given the set of objects O and the empirical distance function on those objects, a weighted undirected graph *G* = {*V*, *E*, *w*} is defined such that V=O, the edges in E capture the relationship between the objects, and *w*_*ij*_ = *f*(*d*_*ij*_) for all pairs of objects $\left(o_{i},o_{j}\right)\in O^{2}$. The function *f* relates the distances to the weights of the graph; it can be simply the identity, but most often it is set to a kernel, such as a Gaussian kernel. Clustering algorithms then assume a certain structure of this graph. In general they assume *k* components with strongly connected objects, the clusters, with weak connections between them [33, 34]. The precise assumptions vary from algorithms to algorithms. Zahn for example constructed a minimum spanning tree on the graph and then detected clusters by deleting the edges with the largest weights [26]. [27, 28] identified clusters based on the idea that they correspond to subgraphs whose vertices are highly connected. This idea was later refined by [30]. Graph partitioning remains however a difficult problem as it relies on the geometry and topology of the graph [35].

Spectral clustering [5, 13] is another class of graph-based clustering algorithms. They first appeared in the early 1970s [36, 37]. The basic idea is still to embed the data points into a graph and identify clusters of points with communities in this graph. The graph is constructed either using an *ϵ* cutoff, a *k*-nearest neighbor graph, or a fully connected graph, with the edges weighted with a Gaussian kernel whose width defines the size of the local neighborhood of the data points [13]. Spectral clustering methods are then based on the spectral analyses of the Laplacian of that graph. The Laplacian matrix is a discrete analog of the Laplacian operator and serves a similar purpose: it measures to what extent a graph differs at one vertex from its values at nearby vertices. The eigen decomposition of this matrix provides a set of eigenvalues and their corresponding orthogonal eigenvectors (corresponding to a basis for the underlying space). If the graph contains *N* disconnected sub-graphs, the eigenvalue 0 appears *N* times, and the corresponding eigenvectors are directly cluster indicators. If the graph is fully connected, the eigenvalue 0 appears once, and its corresponding eigenvector is constant. The following eigenvectors then carry the information about the clusters. This information is retrieved by assigning “coordinates” for the data points based on those eigenvectors. The data points are then clustered based on this new representation, using K-means, or variants of K-means [13]. As expected, this algorithm is now used extensively for detecting communities in graphs (for review, see [35]).

Limitations of spectral clustering however have been highlighted [38]. First, spectral clustering algorithms start from local information encoded in the weighted graph representing the data but generate clusters according to the global eigenvectors of the corresponding Laplacian matrix. The link between local and global features of the data is unclear. Path-based clustering have been proposed for example to capture that link [39–41]. Further, even with a suitable measure of local geometry, a few eigenvectors of the Laplacian matrix cannot successfully cluster datasets that contain structures at different scales of size and density. This problem led to the development of diffusion-based methods that are briefly discussed below.

### Exploring the space of data

The similarity or distance between experimental data points is usually computed by comparing the features describing those data points. As those features can be seen as a vector of real values, distance measures are then maps that compare such vectors, including the Euclidean distance between those vectors, a cosine operator, a correlation coefficient, …Such “empirical” distance measures however do not capture well the actual geometry of the data. For example, the Euclidean distance between points in space would not capture well the geometry of these points if they were embedded on the Swiss roll [4]. To circumvent this problem, one approach is to derive a new distance that is more amenable to describe the geometry. In ISOMAP, this “geodesic” distance is derived by building an *ϵ* graph on the data and generating a new distance matrix based on shortest distance along this graph. The eigen decomposition of this matrix provides a low dimensional embedding of the data that reflects their geometry [4].

The diffusion map algorithm [6, 7] is another method for dimension reduction that relies on the idea of defining a distance that better reflects the geometry of the data. It is anchored in the concept of heat diffusion and random walk Markov chains. The basic idea is that if we take a random walk on the data, walking from one point to a nearby point is more likely to happen than walking to another that is far away. The random walk is not performed explicitly. Instead, a diffusion map algorithm starts by building a kernel *K* on the data, akin to the function *J*_*ij*_ in Eq 1. This kernel is usually set to a Gaussian kernel, with a width *a* that relates to the scale of the data. This kernel is then normalized into a probability matrix, *M*, such that the value *M*(*i*, *j*) between two data points *i* and *j* reflects the probability of walking from *i* to *j* in one step of a random walk. By running the chain forward in time, namely by taking larger and larger powers *t* of *M*, a set of graphs is generated on the data. Eigen decompositions of the Laplacian of those graphs (computed from the powers of M) provides new coordinates for the data points, from which a new distance is computed, the diffusion distance. This family of graphs and related diffusion distances reveal the geometric structure of the data points. While diffusion maps are mainly used for manifold learning [6], they provide information that can provide partitioning of the data into clusters. Indeed, the notion of a cluster in the data set is then quantified as a region in which the probability of escaping this region is low, within a certain time *t*. The drawback of this method is that it requires the computation of multiple powers of the matrix, which may become prohibitive in computing time if the number of data points is large.

## Method

### Basic idea

The DCG++ algorithm takes its inspiration from the different methods described above. It is a graph-theoretic approach; namely, we represent the data points as an undirected weighted graph such that the weights on the edges are functions of the empirical distance on the data, and a temperature scale, *T*. We assimilate this graph to a ferromagnetic potential and use a Monte Carlo algorithm to generate random walks designed to capture the geometry of the data. The result of the random walks is a ensemble matrix, akin to a new adjacency matrix of the graph. The eigen decomposition of the Laplacian of that matrix is used to identify clusters. Information on how the data points are split among those clusters is summarized in the form of a membership matrix. The procedure is then repeated at multiple temperatures, in order to identify the phase transitions of the potential defined on the weighted graph representing the data. The resulting membership matrices are then combined to generate a new distance matrix on the data. We note that this procedure bears similarity with the idea of a diffusion distance computed by the diffusion map algorithms [6], with the main difference that we explore the geometry of the data based on scanning over the parameter defining the local scale of the data, namely the temperature parameter in our approach, rather than scanning the extent with which the random walks are generated, namely the time parameter in the diffusion map algorithms.

The rest of this section provides details on the essential steps of the DCG++ algorithm. Briefly, the clustering method we propose involves four main steps:

(i) Equipping the high dimensional space of the data with a temperature dependent potential energy inspired from statistical mechanics,
(ii) Algorithm 1: At any temperature T, explore the corresponding energy landscape using random walks to generate a graph, and analyze this graph using a spectral clustering approach, and
(iii) Algorithm 2: Repeat steps (i)-(ii) at different temperatures to detect phase transitions, and combine the corresponding information into a new distance matrix that is shown to correspond to an ultrametric.

We note that DCG++ is an extension of previous work in our group. In [14], we had defined a ferromagnetic potential based on the weighted graph representing the data and had proposed a random walk algorithm to explore this potential. The resulting ensemble matrix was then analyzed using spectral clustering to derive a partitioning of the data. In Ref. [15] we proposed to repeat the process at multiple temperatures to generate a new distance matrix on the data points. The whole procedure was named Data Cloud Geometry, in short, DCG. However, DCG had multiple shortcomings, some of which significantly limiting its ability to perform on a large class of data sets. Its major limitation was that the temperatures were considered as parameters that had to be provided as input. In many cases, the range of temperatures and specific values within that range are difficult to define for a specific dataset. Finding a way to define those temperatures automatically from the data was a major driving force behind designing DCG++ described in this paper. In the process of designing DCG++, we have revisited all steps of the DCG procedure. In the following, we describe the new algorithms and justify the changes that were implemented.

### Algorithm 1: Partitioning the data at one scale (temperature) T

#### Exploring the data at a temperature T

Let *S* = {*s*_1_, *s*_2_, …*s*_*N*_} be the set of *N* data points considered, and let *d* be the empirical distance measure on *S*, assumed given as input. This distance *d* is usually computed by comparing features of those data points, either using a Euclidean distance between those features, or a correlation coefficient, or any other measure of dissimilarity between vectors. For sake of generality, we do not assume that *d* is a metric on *S*. Instead, we only assume that it corresponds to a positive, symmetric kernel, namely that it satisfies the following two properties for all (*s*_*i*_, *s*_*j*_) ∈ *S*^2^,

$$\begin{array}{ccc} d(s_{i},s_{j}) & \geq & 0\\ d(s_{i},s_{j}) & = & d(s_{j},s_{i}) \end{array}$$

We then define a kernel on the data points:

$$W\left(s_{i},s_{j}\right)=\{\begin{array}{cc} \text{exp}(\frac{-d(s_{i},s_{j})}{T}) & \;s_{i}\neq s_{j}\\ 0 & \;s_{i}=s_{j} \end{array}$$ (2)

*W* is also a positive symmetric kernel. It constitutes our definition of the local geometry of *S*, captured by the “scale” parameter *T*, which we refer to as a temperature (this will be explained in the next subsection). We then construct a fully connected weighted undirected graph *G* = {*V*, *E*, *w*} on the data such that *V* = *S*, the edges in *E* include all pairs (*s*_*i*_, *s*_*j*_), and the weight *w*(*s*_*i*_, *s*_*j*_) = *W*(*s*_*i*_, *s*_*j*_) for all pairs of objects (*s*_*i*_, *s*_*j*_) ∈ *S*^2^. Setting *W*(*s*_*i*_, *s*_*i*_) to zero therefore refers to removing self-edges in this graph.

From the graph *G*, we can construct a reversible Markov chain on *S*. Let us set

$$D\left(s_{i}\right)=\sum_{j=1}^{N}w\left(s_{i},s_{j}\right)$$

to be the weighted degree of vertex *s*_*i*_ and let us define:

$$K_{T}\left(s_{i},s_{j}\right)=\frac{w(s_{i},s_{j})}{D(s_{i})}$$ (3)

We write *K* with subscript *T* to indicate that it is a function of *T*. *K* keeps the positive property of *w* but it is no more symmetric. However, *K*_*T*_ does satisfy the conservation property

$$\sum_{j=1}^{N}K_{T}\left(s_{i},s_{j}\right)=1$$

for all vertices *s*_*i*_ in *V*. *K*_*T*_ can therefore be viewed as the transition kernel of a Markov chain on *S*. In other words. *K*_*T*_(*s*_*i*_, *s*_*j*_) is interpreted as the probability *p*(*s*_*i*_, *s*_*j*_) of transition from *s*_*i*_ to *s*_*j*_ in one step at a given scale *T*. For t∈N, let *p*^(*t*)^(*s*_*i*_, *s*_*j*_) represent the probability of transition in *t* time steps from *s*_*i*_ to *s*_*j*_; note that *p*^(*t*)^ is the kernel associated to the matrix $K_{T}^{t}$. As shown in [42], running the Markov chain forward, or equivalently taking powers of *K*_*T*_, reveals relevant geometric structures of *S*. In particular, small powers of *K*_*T*_ will segment the data set into several smaller clusters, while at larger time *t* the clusters evolve and merge together until in the limit as *t* → ∞ the data set is grouped into one cluster.

To compute the transition probabilities after exactly *t* steps of the Markov chain, we can either directly compute $K_{T}^{t}$, or explicitly perform random walks with *t* steps starting from each of the vertices in the graph. The former solution involves multiple matrix products; as we do not filter (i.e. we do not apply a cutoff that sets small values in the matrix to zero) the transition matrix *K*_*T*_, it is dense and therefore the complexity of computing one matrix multiplication is *O*(*N*^3^). We note that there are faster algorithms for matrix multiplication, such as the Strassen algorithm originally introduced as early as in 1969 [43]; we did not implement any of those algorithms and relied instead on the BLAS implementation of matrix multiplication as it is readily parallelized [44]. Computing $K_{T}^{t}$ has then a complexity of *O*(*tN*^3^), which can be prohibitive for large *N* and *t*. We have therefore implemented the latter solution, namely computing the random walks explicitly. Each random walk starts from a seed vertex *s*_*k*_ and continues for *t* steps, with the probability of jumping from a vertex *s*_*i*_ to a vertex *s*_*j*_ along the walk set to *K*_*T*_(*s*_*i*_, *s*_*j*_). *P* independent random walks are performed for each *s*_*k*_. For each *t*-step random walk starting at *s*_*k*_, we accumulate the number of visits to vertices *s*_*i*_ as *V*(*k*, *j*). The output of this process is an ensemble matrix *E*_*T*_ defined by:

$$E_{T}\left(i,j\right)=\frac{\sum_{p=1}^{P}\left(V\left(i,j\right)+V\left(j,i\right)\right)}{2Pt}$$ (4)

The matrix *E*_*T*_ is a symmetric approximation of $K_{T}^{t}$. Note that the complexity of computing *E*_*T*_ is *O*(*PtN*). As *P* is usually taken of order *log*(*N*), while *t* is of order *N* (see below), this leads to a complexity of order *O*(*N*^2^ *log*(*N*)), i.e. a significant improvement compared to the *O*(*N*^3^) complexity for computing directly $K_{T}^{t}$.

**Differences with DCG** The original implementation of the data exploration with DCG [14] follows a similar algorithm, with two significant differences. First, in the original DCG the random walks are performed with removal of vertices, once those vertices have been visited frequently. We found however that this vertex removal led to problems when trying to capture non convex geometries. Second, the ensemble matrix was computed by monitoring the energy along the walk, and identifying significant changes in energy due to the system jumping from one local minimum of the potential to another. Finding the threshold to use to characterize these “significant” changes in energy value proved to be a problem for large, well-connected sets of data points. The procedure described here follows a more traditional random walk approach.

#### Partitioning the graph based on the ensemble matrix

The ensemble matrix *E*_*T*_ defines a new set of weights for the graph representing the data points, that are expected to better capture the geometry of that graph than the original weight matrix *W*. Given this new weight matrix, we compute a partitioning of the graph using a modified version of the spectral clustering algorithm proposed by Ng and colleagues [5]. We first compute the normalized symmetric Laplacian of the graph from its weighted adjacency matrix *E*_*T*_:

$$L_{T}=I-B_{T}^{-\frac{1}{2}}E_{T}B_{T}^{-\frac{1}{2}}$$ (5)

where *B*_*T*_ is the diagonal degree matrix of *E*_*T*_, i.e. $B_{T}\left(i,i\right)=\sum_{j=1}^{N}E_{T}\left(i,j\right)$ and *B*_*T*_(*i*, *j*) = 0 when *i* ≠ *j*. We assume that the number of clusters corresponding to *E*_*T*_ is between 1 and *M*, where *M* is considered to be sufficiently larger than the actual number of clusters *K*(*T*). We then compute the *M* smallest eigenvalues of *L*_*T*_, Λ = (λ_1_, …, λ_*M*_), and the corresponding eigenvectors *V* = (**v**_**1**_, …, **v**_**M**_). The eigenvalues are given in non-decreasing order. Note that they are all expected to be between 0 and 1. The actual number of clusters *K*(*T*) is then set to the number of eigenvalues that have a magnitude smaller than a prescribed threshold *C*. The data point *s*_*i*_ is then assigned a set of *K*(*T*) coordinates, *X*(*i*, *k*), such that:

$$X\left(i,k\right)=\frac{v_{k}\left(i\right)}{(\sum_{j=1}^{K(T)}v_{j}{\left(i\right)}^{2}{)}^{1/2}}$$ (6)

where *v*_*k*_(*i*) is the i-th component of the eigenvector **v**_**k**_. The *N* data points in *S*, represented at this stage in a *M*-dimensional space with coordinates defined above are then partitioned into *K*(*T*) clusters using the K-means++ algorithm [45]. The procedure is repeated *k* times, using different seed centers for the cluster, and the partitioning that gives the smallest sum of variances is selected. This result of this partitioning is then stored as a binary membership matrix, denoted as $M_{T}$, with $M_{T}\left(i,j\right)=1$ if *s*_*i*_ and *s*_*j*_ are found to be in the same cluster and $M_{T}\left(i,j\right)=0$ otherwise.

**Differences with DCG** Compared to the original implementation of the data exploration with DCG [14], we only compute the top *M* eigen pairs of the symmetric, normalized Laplacian to reduce the computing time, and use an automatic selection of the number of clusters, based on the threshold *C*.

### Algorithm 2: Exploring the energy landscapes at multiple temperatures

Algorithm 1 described above rests on two main parameters: the scale, or temperature *T* that defines the size of the neighborhood around each point, and the time *t* for the Markov chains that explore the weighted graph whose adjacency matrix is based on *T*. In diffusion map algorithms, it is argued that *T* is a characteristics of the data that should be considered as given, while the time *t* is a reaction coordinate that enables exploration of the geometry of the data. DCG++ is based on a dual concept. We assimilate the weighted graph to a potential landscape, typically characterized by many wells with various depths. A random walk on this landscape will identify the many wells of the potential, as well as the probability of jumping from one well to another. At a high temperature *T*, the walk will transition from any points to most of the other points with more or less equal probabilities: the graph will be seen as complete, with a single cluster. At a low temperature however, the Markov chain tends to get trapped in potential wells for various periods of time depending on the sizes of the wells before it can escape. The analysis of the Markov chain will then result in the detection of many clusters. The temperature *T* becomes then a reaction coordinate that allows us to detect the multiple scales of the geometry of the data. Algorithm 2 in DCG++ implements the exploration of this reaction coordinate *T* in an automated manner. Starting with the empirical distance matrix *d*, and a prescribed number of clusters *M*, where *M* is considered to be sufficiently larger than the actual number of clusters *K*(*T*), algorithm 2 proceeds in three steps:

i) ***Set the lower limit for*** *T*, *T*_0_. As described above, at a low temperature, the data are expected to be partitioned into a large number of clusters. We initialize *T* to the average nearest neighbor distance among all pairs of points [16]. We then apply algorithm 1 with this temperature; if the number of clusters detected is smaller than the prescribed value *M*, *T* is decreased by a factor 2. The procedure is then repeated until the number of clusters is at least *M*, in which case *T*_0_ is set to the current *T*. The corresponding number of clusters may be larger than *M*, in which case *M* is updated to that value.
ii) ***Set the upper limit for*** *T*, *T*_*max*_. At a high temperature, the data are expected to belong to a single cluster. We initialize *T* to be twice *T*_0_ and apply Algorithm 1. If the number of clusters is larger than 1, we double *T* again, and reapply algorithm 1. This procedure is repeated until the number of clusters detected is 1, in which case *T*_*max*_ is set to the current *T*.
iii) ***Find the transition temperatures between*** *T*_0_ **and** *T*_*max*_. As the temperature increases from *T*_0_ to *T*_*max*_, the geometry of the graph representing the data will change, with clusters progressively evolving and merging until a single cluster remains. As the transitions are revealed at discrete values for the number of clusters, these transitions are more step functions than smooth functions. We reveal those transitions with a simple bracketing procedure based on a binary search. For a given expected number of clusters *k*, we initialize the bracket [*T*_*low*_, *T*_*high*_] based on information from the search at *k* − 1 (for example, for *k* = 2, [*T*_*low*_, *T*_*high*_] = [*T*_0_, *T*_*max*_]). We then set *T*_*try*_ to the middle of the bracket. If the number of clusters identified by algorithm 1 for *T*_*try*_ is equal to *k*, *T*_*try*_ is stored and we move to the next value of *k*. Otherwise, *T*_*low*_ or *T*_*high*_ are updated to *T*_*try*_, and the procedure is iterated until the number of clusters matches with *k*, or when the size of the bracket goes below a threshold *ϵ*. In the former case, the temperature is recorded, while in the latter case, no temperature is recorded for that value of *k*. The procedure is then repeated until *k* = *M* − 1. Note that the total number of recorded temperatures may be smaller than *M*, as the procedure may have “failed” for some specific cluster number, when the bracket interval becomes too small.

The output of this algorithm is a set of temperatures, one at each change in the number of clusters (see above). To improve the sampling of the transition curve, we add to this set intermediate temperatures, set at the midpoints of the consecutive temperature intervals. This leads to a new set *ST* = {*T*_0_, *T*_1_, …, *T*_*max*_} such that |*ST*| ≤ 2*M* + 1.

**Differences with DCG** The initial version of DCG selected the transition temperatures manually. We observed that this lead to a crude representation of the full transition. We therefore designed algorithm 2 to provide an automatic selection of those temperatures.

### Generating a new distance matrix on the data

Algorithm 1 is then run for each temperature *T*_*k*_ in the set *ST* generated by algorithm 2. Each of these runs leads to a binary membership table $M_{T_{k}}\in R^{N\times N}$. These membership tables are then combined into a matrix *U* as follows.

Recall that the entry (*i*, *j*) of ensemble matrix $M_{T_{k}}$ indicates whether the data point *s*_*i*_ and *s*_*j*_ were found to belong to the same cluster at temperature *T*_*k*_. For each pair of points (*s*_*i*_, *s*_*j*_), we have then a sequence of indicators that the points are co-clustered over the range of temperatures, $\left\{M_{T_{0}}\left(i,j\right),M_{T_{1}}\left(i,j\right),..M_{T_{k}}\left(i,j\right)\right\}$. We construct a matrix $U\in R^{N\times N}$ whose entries record from which temperature two points consistently belong to the same cluster. Namely,

$$U\left(i,j\right)=\text{min}\{T_{k}\;|\;\prod_{l=k}^{K}M_{T_{l}}\left(i,j\right)>0\}$$ (7)

Note that the cluster-sharing sequence for a pair of points (*s*_*i*_, *s*_*j*_) may contain more than one switch from zero to one. Such repeated switches can be seen as noise, not unexpected due to the heuristic nature of the random walks in algorithm 1 (among other possible sources of noise). Eq 7 handles this noise by selecting the last 0-to-1 switch. This scheme also leads to the matrix *U* corresponding to an ultrametric. Finally, the matrix *U* is scaled with a monotonic increasing linear transformation so that its elements fall in the interval [1, 100], where 100 is chosen arbitrarily to spread the distance values over a wide range.

**Proposition 1**. *The DCG*++ *generated distance matrix U is an ultrametric distance matrix*.

*Proof*. Given a set of *N* points *S* = {*s*_0_, …, *s*_*N*−1_}, and a matrix distance *D* on *S*, *D* is said to be an ultrametric distance if it satisfies the three conditions for all (*i*, *j*, *k*) ∈ [1, *N*]^3^:

(1) *D*(*i*, *j*) ≥ 0
(2) *D*(*i*, *j*) = *D*(*j*, *i*)
(3) *D*(*i*, *j*) ≤ max(*D*(*i*, *k*), *D*(*j*, *k*)).

We note first that the distance matrix *U* generated by DCG++ satisfies conditions (1) and (2) by construction, as the temperatures *T*’s are positive, and all the membership matrices $M_{T}$ are symmetric, resulting in *U* being symmetric.

We prove condition (3) for *U* (the strong triangular inequality), namely that *D*(*i*, *j*) ≤ max(*D*(*i*, *k*), *D*(*j*, *k*)) for any three points with indices *i*, *j*, and *k* in *S*. let us define *T*_*ij*_ = *U*(*i*, *j*), *T*_*ik*_ = *U*(*i*, *k*), and *T*_*jk*_ = *U*(*j*, *k*). By definition of the matrix *U* (see Eq 7), we have:

$$\forall T\geq T_{ik}\;\;M_{T}\left(i,k\right)=1$$

and a similar property for *T*_*jk*_. Therefore, ∀*T* ≥ max(*T*_*ik*_, *T*_*jk*_), we have $M_{T}\left(i,k\right)=1$ and $M_{T}\left(j,k\right)=1$. This means that ∀*T* ≥ max(*T*_*ik*_, *T*_*jk*_), the three data points *s*_*i*_, *s*_*j*_, and *s*_*k*_ are found to belong to the same cluster with algorithm 1. In particular, (*s*_*i*_, *s*_*j*_) are in the same cluster for all those *T*; using again the definition of the matrix *U*, we have *T*_*ij*_ ≤ max(*T*_*ik*_, *T*_*jk*_). Replacing the *Ts* with their definitions with respect to *U* validates condition (3), which then concludes the proof.

## Implementation

DCG++ was designed as a stand-alone applications written mainly in C++, with some calls to libraries in Fortran (see below). The source code of DCG++ is available at https://github.com/pkoehl/DCG. Here we briefly describe some specifics of the implementation and review all the parameters that need to be set when running the program.

For a data set with *N* points, algorithm 1 starts with performing *N* × *P* random walks, where *P* is the number of independent repeats for one point, each of length *t*, and *t* is the time, corresponding to the number of steps. As the *N* × *P* walks are independent from each other, their computations can be trivially parallelized. We have used the standard pthread library from C to implement this parallelization.

Once the random walks have been completed, algorithm 1 proceeds by generating the normalized symmetric Laplacian *L*_*T*_ of the corresponding ensemble matrix and computing the eigen decomposition of this matrix. We note that we do not need to compute the full spectrum of this eigen decomposition, which may be prohibitively expensive when the number of points *N* is large. Instead, we only compute a small number of the eigenpairs, those corresponding to the eigen values with smallest magnitude. Those eigen pairs can be efficiently computed using a Lanczos method [46]; we have used the Fortran package ARPACK [47] for this task.

In addition to the data points and the empirical distance measure on those data (provided either directly in the form of a distance matrix, or with the data characterized by features and an option for computing the distance between those features, such as Euclidean distance, correlation distance, or Hamming distance for binary data), DCG++ requires input for the values of its parameters. Random walks are characterized by two parameters, the number *P* of randoms walks for each data point, and the number of steps *t* in each random walk. Defaults values for those parameters are set to *P* = 5, and *t* = *N*, where *N* is the number of data points (although smaller values for *t* are often used when *N* is large, see the experimental analyses below). In algorithm 1, the cutoff values for defining the number of clusters *C* and the number of repeats for the Kmeans++ algorithm are set to 0.2 and 50. Finally in algorithm 2, the tolerance *ϵ* for the bracketing procedure is set to 0.005. Those values have been found to work well for the test cases presented below.

## Experimental analysis

### Experimental setting and assessment measures

We validate and verify the effectiveness of the DCG++ algorithm on several synthetic and real data sets. The synthetic datasets came from the “Clustering basic benchmark” http://cs.joensuu.fi/sipu/datasets/ while the real datasets were downloaded from the UCI Machine Learning repository https://archive.ics.uci.edu/ml/index.php. As DCG++ is designed to generate a new (ultrametric) distance on the given data points based on an empirical distance, it is not a clustering algorithm per se. Therefore, the validations focus on the improvements that the new distance may induce, compared to using directly the empirical distance provided with the data. Such validations are performed using two assessment tools, namely a Receiver Operator Characteristics (ROC) analysis, and classification experiments.

**ROC analysis** We quantify the effectiveness of a distance measure in identifying correctly that two data points belong to the same cluster using the ROC analysis. A pair of points is defined as similar, or “positive”, if they belong to the same cluster, and “negative” otherwise. All pairs of points in a dataset are then compared using a similarity measure. For varying thresholds of the measure, pairs whose corresponding distance falls below the threshold are assumed positive, and all above it are negative. The pairs that agree with the standard are called true positives (TP), while those that do not are false positives (FP). ROC analysis compares the rate of TP as a function of the rate of FP; it is scored with the Area Under the corresponding Curve, namely the AUC. An AUC score of 1 indicates that all TP are detected first: this corresponds to an ideal measure. On the other hand, an AUC score of 0.5 corresponds to the first diagonal: TP and FP appear at the same rate, and the measure is not discriminative.

**Classification experiments** The ROC analysis described above ranks distances between data points and assesses if this ranking is compatible with an existing classification; it does not perform the classification itself. We extend the ROC analysis to the actual problem of pattern recognition by performing a second set of computational experiments. Each experiment involves a data set of points, *D* and a distance measure, *d*. We begin by randomly dividing the sets of points in *D* into two groups of approximately equal size. The first group serves as a training set, while the second group serves as a test set. A test point is classified by assigning it to the nearest cluster in the training set. Here nearest cluster is defined in two different ways. It is either the cluster of the training point that is closest to the test point (“single linkage”), or it is obtained by computing first the mean distance between the test point and all points in the training set that belongs to a given cluster, for all clusters, and then taking the smallest of those mean distances (“average linkage”). The results are stored in a confusion matrix, *C*, whose element *C*(*i*, *j*) reports the number of points that belong to cluster *i* but have been classified as belonging to cluster *j*. The accuracy of the distance *d* as a classifier is then defined to be the ratio of the trace of the confusion matrix over the sum of all its elements (i.e. the percentage of correctly classified data points). To remove the influence of the initial division of the data set into test and training sets, the procedure is repeated 10000 times.

Results are also presented visually, using hierarchical clustering based on the two distance matrices, the Empirical Distance and Ultrametric distance. We will use the acronym ED-HC and UD-HC when referring to the former and latter, respectively.

All experiments were conducted on an Apple computer with an Intel i7 4GHz processor with 4 cores and 64 Gb of RAM.

### A toy problem: The spiral test case

**Fig 1:**
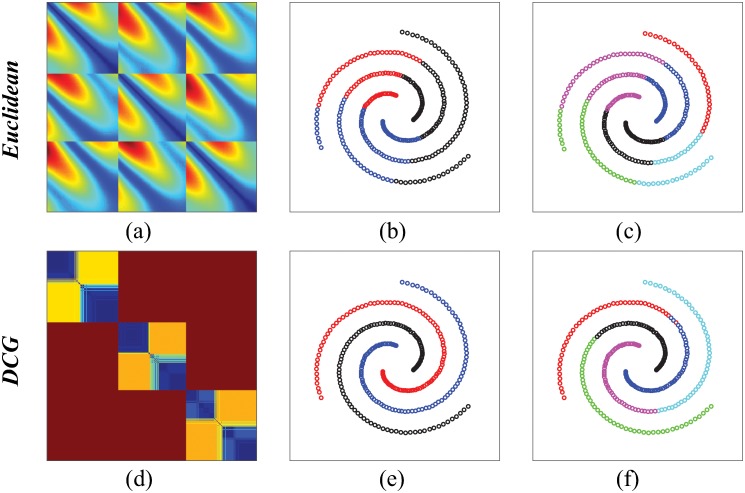
Clustering the 3-spiral data set. The spiral dataset includes 312 points, partitioned into three spirals, each with 104 points. (a) The Euclidean distance matrix for the 312 points, ordered by partition ID. We see structures within each partition, but also significant interactions between the partitions. Hierarchical clustering with Ward linkage is applied on this matrix; the corresponding tree is cut at 3 clusters, (b), and 6 clusters (c), respectively. The clusters are compact and do not map with the actual spirals. (d) The ultrametric matrix *U* derived by DCG++. The three partitions are clearly identified, with additional structures within each group. Hierarchical clustering with Ward linkage is applied on this matrix; the corresponding tree is cut at 3 clusters, (e), and 6 clusters (f), respectively. The clusters map with the spirals.

The first test set we consider is the 3-spiral data set (see Fig 1) [41]. This data set is not unusually complex, as the concept of clusters is well defined, with 3 clusters corresponding to the 3 spirals. It highlights however the possible shortcomings of the similarity measure used to compare the points. While the Euclidean distance is a natural metric for comparing the positions of points on the plane, it does not capture the geometry of the spirals, as illustrated in Fig 1(a). Indeed, while short distances correspond to the local neighborhood within a spiral, medium and long distance values are not discriminative, i.e. two points whose Euclidean distance is large have the same probability to be on the same spiral than to be on different spirals. Hierarchical clustering based on this Euclidean distance leads to compact clusters that partition the plane into convex regions that do not match with the spirals, as illustrated in Fig 1(b) and 1(c). Applying the DCG procedure described here does correct the shortcomings of the Euclidean distance. DCG++ was run with *t*, the number of steps in the random walks set to 700, *P*, the number of random walks per point set to 5, and the upper limit to the number of clusters set to 10. In Fig 1(d), the heatmap for the ultrametric distance *U* clearly identifies three main clusters, with internal structures within those clusters. In Fig 1(e) and 1(f), we show that the three clusters observed on the heat map map to the three spirals, while the internal structures within the clusters lead to partitioning of the spirals themselves, each with the same number of sub clusters. We note that DCG++ is not the only solution for analyzing this data set correctly. The ISOMAP procedure for example was designed to circumvent the same deficiencies of the Euclidean distance measure [4], while path-based spectral clustering improved upon using a simple modification of the Euclidean distance with a Gaussian kernel [41]. DCG++ is an equivalently easy procedure to implement, with a broader range of applications, as illustrated below.

### A second toy problem: Two overlapping clusters

**Fig 2:**
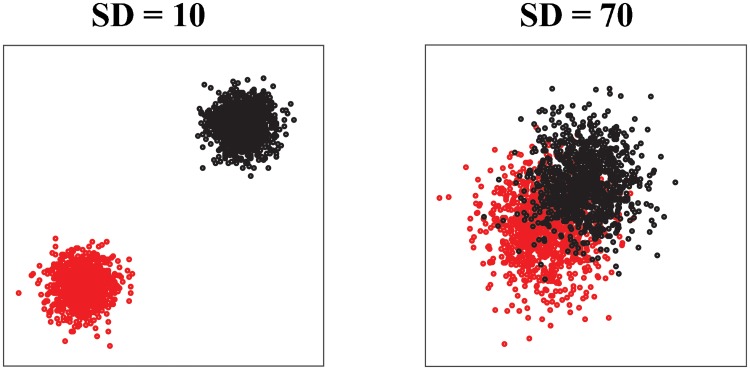
Two clusters based on two 2D Gaussian distributions with varying widths.

**Fig 3:**
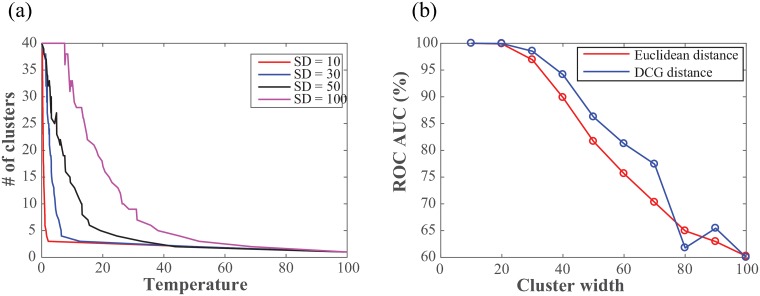
Analyzing the two-cluster dataset at varying level of overlap. (a) The transition curves that relate the number of clusters found in the dataset to the scale defining the local geometry are shown for varying values of the width of the Gaussian distributions defining the clusters. (b) We compare the efficiency of the Euclidean distance (red) and the ultrametric distance *U* to detect cluster memberships as a function of the cluster width *SD*. Results are derived from ROC analyses and reported as AUC values, with large and small AUC values corresponding to good and poor discrimination, respectively.

The second test set we consider is again academic. It includes two clusters whose points have been generated based on 2D Gaussian distributions, with varying widths *SD* (see Fig 2 and [48]). Those clusters are compact, as opposed to the non-linear geometry of the spirals considered above; however the concept of clusters itself becomes more difficult to discern, as those clusters show significant overlaps for large values of *SD*. For each value of *SD*, there are 2048 points total, 1024 per cluster. We have run *DCG*+ + with *t* = 100 steps per random walk, *P* = 5 repeats for each data point, and a upper limit of 40 for the number of clusters. We have tested whether the Euclidean distance, or the ultrametric distance U can identify cluster membership at varying amounts of overlaps using ROC analysis. Results are shown in Fig 3.

The DCG++ algorithm works by assessing the partitions of the data when the “temperature” or scale of its kernel increases, i.e. as we change the definition of the local geometry. In Fig 3(a), we plot the number of the partitions *N*, found as a function *f*(*T*) of the local scale, for different versions of the two cluster datasets. When *SD* is small (10 or 30), the two clusters are well separated and *f*(*T*) shows a clear transition between 2 and 3 clusters. When *SD* is large however (70 or 100, i.e. close to the distance between the centers of the clusters), the transition curve *f*(*T*) is noisier and does not allow for a clear definition of a number of clusters.

As expected, the curves *AUC* = *f*(*SD*) for both the Euclidean distance and ultrametric distance *U* are monotonically decreasing (see Fig 3(b)): for small values of *SD*, the clusters are well separated and a small distance is a good indicator of cluster membership, while at large values of *SD* the two clusters overlap and distances are no more discriminative. These two curves differ however for “medium” values of *SD*, in which case the ultrametric distance is seen to provide a better detection of cluster membership.

### Synthetic data: Clusters with complex geometry

**Fig 4:**
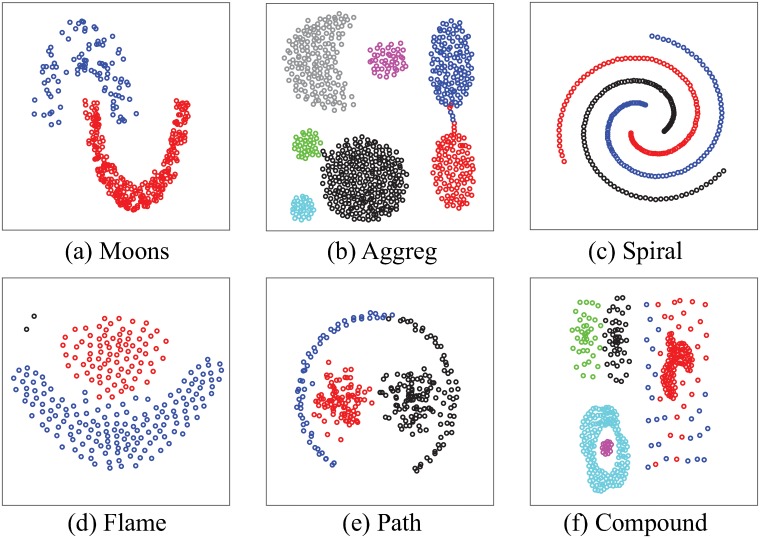
Test cases with clusters with complex geometries (see text for details). All these datasets were obtained from https://cs.joensuu.fi/sipu/datasets/.

Our next benchmark involves multiple datasets representing clusters with complex geometries, as illustrated in Fig 4. The two moons dataset [49] is a standard toy problem used to assess clustering techniques on non-convex clusters, the aggreg dataset [50] includes compact clusters of various sizes and various inter cluster distances, the flame dataset [51] is similar in difficulty to the moons data set, with the additional presence of two spurious data points, the compound [26] dataset includes clusters within cluster, while the path [41] dataset was designed to test path-based spectral clustering techniques. We added the 3-spiral dataset already discussed above, as it fits with this set of data with complex geometry. For each dataset, we computed two distance matrices over all the points they contain, namely the Euclidean distance and the ultrametric distance *U* derived by the DCG procedure. The latter was computed with *t* = 1000 steps per random walk, *P* = 5 repeats for each data point, and an upper limit of 20 for the number of clusters. We then analyzed how well those distance matrices capture the partitioning of the data, using a ROC analysis, as well as a set of computational classification experiments.

The ROC analysis we have implemented is designed to assess the extent with which a distance between data points reveals if those points belong to the same cluster. The area under this curve, AUC, should be large for distances that map well with cluster membership, and small otherwise. For the Euclidean distance, the AUC is expected to be large when the clusters are compact. Indeed, it is found to be 98% for the aggreg dataset, Table 1. The AUC however is lower when the geometry of the cluster is not convex, reaching 50%, i.e. the expected value for a random assignment of data points to clusters, for the spiral data set. In contrast, the ultrametric distance computed with DCG performs consistently well, with large AUC values above 98%, independent of the geometry of the cluster (Table 1). We do note however one exception, the path dataset. For this dataset, the ultrametric distance does lead to an improvement compared to the Euclidean distance, but not to the same extent as what is observed with the other test cases (with an improvement in AUC of 10% for the path dataset, compared to improvements of 25% and 50% for the Flame and spiral datasets, for example). It is unclear at this stage why this is the case.

**Table 1:** Euclidean distance vs DCG distance for detection partitions in datasets with complex geometries.

	Euclidean Distance			Ultrametric DCG Distance		
Dataset	AUC ^a^	Single-CA ^b^	Ave-CA ^c^	AUC ^a^	Single-CA ^b^	Ave-CA ^c^
Two moons	80.0	100.0 (0.05)	88 (2)	100.0	100.0 (0.3)	100.0 (0.3)
Aggreg	98.0	100 (0.2)	94 (1.1)	99.5	99.5 (0.4)	93.5 (1.1)
Spiral	50.0	100.0 (0.5)	46.1 (8.4)	100.0	100.0 (0)	100.0 (0)
Flame	73.3	99.0 (0.9)	86.2 (2.4)	98.0	99.2 (1.0)	99.0 (0.9)
Path	72.6	99.1 (0.8)	64.4 (1.0)	84.0	98.0 (1.1)	63.6 (0.4)
Compound	94	97.3 (0.9)	50.6 (2.2)	99.2	94.0 (1.2)	90.6 (1.5)

^a)^ Area Under the Curve, AUC, in percent based on ROC analysis of the power of the distance as an indicator of cluster membership. The higher the AUC, the better the distance is.

^b)^ Accuracy (in percent) when the distance is used for classification, with a single linkage for assigning a test point to a training cluster; standard deviation based on 10000 classification experiments is provided in parenthesis. The higher the accuracy, the better the distance is for classification purpose.

^c)^ Accuracy (in percent) when the distance is used for classification, with an average linkage for assigning a test point to a training cluster; standard deviation based on 10000 classification experiments is provided in parenthesis.

The ROC analysis described above detects similarity. We extended to the problem of detecting partition in the data by performing a set of computational classification experiments (see subsection above). The results of those experiment are stored in a confusion matrix. The accuracy of the distance as a classifier is then defined as the ratio of the trace of that matrix over the number of test points, i.e. the percentage of correctly classified points. This classification accuracy (CA) is named Single-CA and Ave-CA for the single linkage and average linkage experiments, respectively. Results for both the Euclidean distance and ultrametric distance are given in Table 1. The Euclidean distance is found to be an accurate support for classification when the single linkage is used. This is expected, as local distance reflects cluster membership, even for the complicated geometry of the 3-spiral dataset. When the average linkage is used however, the classification accuracy based on the Euclidean distance drops significantly for the data set with complex geometry. In contrast, the ultrametric distance leads to accurate classification for both linkages. We do note that we observe the same exception than for the ROC analysis, namely the path dataset. We are currently exploring why this is the case.

For illustration purpose, we show in Fig 4 the results of clustering the different data sets using hierarchical clustering based on the Ultrametric distance with the Ward linkage, with the resulting tree cut at the actual number of clusters in the data set (except for the flame dataset, see below). All resulting clusterings match with visual intuition, with the known exception of the path dataset (see above). For the flame dataset, we cut the hierarchical tree at 3 clusters. The two main clusters remain unaltered compared to a cutting at two clusters, and the two spurious point on the left top corner of the figure form a cluster on their own.

### Real data: Clustering frogs based on their vocalization capability

Real data differ significantly from the test problems described above. Their data points usually do not have “coordinates” and cannot be displayed directly. Instead, they are characterized by features, in many cases a large number of them. The definition of a distance measure for comparing those features is then not as straightforward as using the Euclidean distance when comparing the positions of two points in a Euclidean space. The choice of the distance is not universal, and depends on the specifics of the data considered. In this section, we compare such a proposed empirical distance measure with its modified ultrametric version derived by DCG++ on a dataset of time series that capture the calls of anurans.

Amphibians are directly affected by changes in the environment [52, 53]. Many scientists then monitor the decline in amphibian populations and use it as an indicator of environmental problems. The most studied species in that regard are anurans (frogs and toads). Scientists take advantage of their vocalization capability and apply acoustics surveys to identify their numbers. Interestingly, time series of their calls have also been used to identify families, genera, and species among those anurans [54]. In fact, there is a large body of literature on that topic that goes beyond the scope of this paper. We consider one data set of such time series, available at the UCI Machine Learning Repository https://archive.ics.uci.edu/ml/index.php under the name MFCC.

The dataset was created by segmenting 60 audio records belonging to 4 different families, 8 genus, and 10 species of anurans. Each record corresponds to one frog. These records were collected in situ, in South America. From the segmentation of those record, 7195 syllables were derived, which form the data points for our analysis. Each syllable is then characterized by 22 features, Mel-Frequency cepstral coefficients (MFCC, hence the name of the dataset). These MFCCs have been normalized between -1 and 1. The family, genus, and species assignments are known for each of those syllables, and used for assessment. We consider two distances on those syllables, namely the Euclidean distance between their MFCCs, and the ultrametric distance *U* derived from the Euclidean distance with the DCG algorithm. DCG++ was run with *t* = 500 steps in the random walks, *P* = 5 repeats for data point, and a upper limit of 20 for the number of clusters. These distance measures were then assessed in their ability to identify families, genera, and species using the ROC analysis.

**Fig 5:**
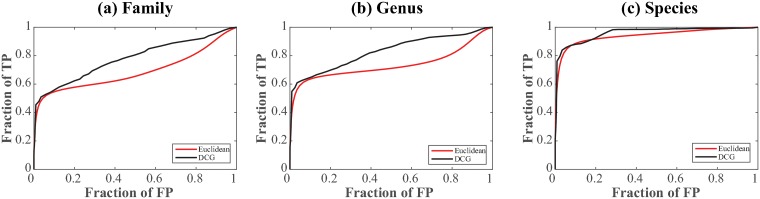
ROC analyses of two measures of sound similarity. We compare the efficiency of two distances, the Euclidean distance and the Ultrametric DCG-derived distance, to detect similarities between frogs at the family (a), genus (b), and species (c) level. Curves close to the first diagonal indicate poor performance, while the curves that are close to the top right corner indicate good performance.

Fig 5 compares the ROC curves derived from the Euclidean distance and DCG ultrametric *U* at three levels of classification of the syllables, family (a), genus (b), and species (c). The AUC for the Euclidean distance are 69%, 73%, and 94% at the family, genus, and species levels, respectively, while the corresponding AUC for the ultrametric distance are 77%, 83%, and 96%, revealing a consistent improvement induced by DCG at all three levels. The ROC curves illustrate that short distances (both Euclidean and DCG) map well with the partitioning of the frogs at all three levels. The differences between the Euclidean distance and ultrametric distance become apparent for larger distances, especially at the family and genus levels. Medium to large Euclidean distances are less discriminative, a fairly common problem of empirical distances. In contrast, the ultrametric distance consistently performs better for this range of distances.

### Image segmentation

**Fig 6:**
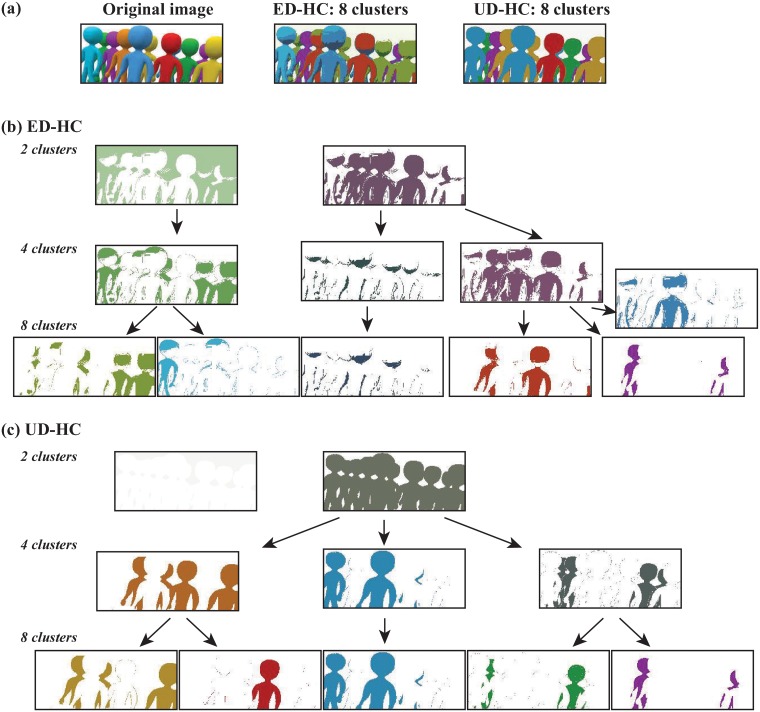
Comparative image segmentation using two distance measures. (a) The original image, and the reconstructed images obtained by partitioning the pixels of the image using hierarchical clustering with complete linkage, based on the Euclidean distance (ED-HC), and the DCG derived ultrametric distance *U*, UD-HC. The images at different levels of the hierarchical trees computed from the Euclidean distance (b), and ultrametric distance (c). We focus on the segmentation of the different robots. For clarity, we omit at the 4-cluster and 8-cluster levels the images representing clusters that only contain pixels related to background.

Our last benchmark is an image segmentation problem. Analysis is performed based on visual inspection only, as the “ground truth” is not available. We consider a small image of ten stylized characters standing next to each other (see Fig 6(a)). Those characters appear in different colors, blue, purple, orange, red, and green with some variations within some of those colors (for example the three blue characters show different intensities of blue). The image is size 205x104, i.e. it contains 26,000 pixels. Each pixel is defined by three coordinates in the L*a*b (i.e. Lightness, L, and two color components a and b capturing green-red and blue-yellow, respectively) color space. We have used the MATLAB function rgb2lab to generate those coordinates from the original image in the RGB color space. The image is therefore represented with 26,000 “objects”, the pixels, and three coordinates per object. We compared and partitioned those pixels using two distance measures, the Euclidean distance, and the DCG-derived ultrametric. The latter was computed with DCG++, with *t* = 20, 000 step in each random walk, *P* = 10 repeats for each point, and a upper limit of 20 for the number of clusters. The corresponding distance matrices were then given as input to a hierarchical clustering procedure (from MATLAB), with complete linkage. The resulting trees were cut at 2 clusters, 4 clusters, and 8 clusters. At each level, pixels belonging to the same cluster were assigned the same color, computed as the average of their color coordinates in the original image. Results of those analyses are given in Fig 6.

The fully reconstructed images at the 8 cluster levels based on both distance measures resemble the original images, as shown in Fig 6(a). However, the image reconstructed from the DCG-based distance is more faithful with respect to its identification of the different types of robots included in the image, and therefore its restitution of the colors of the original image. When we follow the different levels of the hierarchical trees, differences between the Euclidean distance and DCG distance becomes visually more striking. At the 2 cluster level, the partitioning derived from the DCG distance clearly maps with a separation of the characters and background in the image. In contrast, the Euclidean distance based partitioning shows two clusters that both contain characters and background (Fig 6(c)). As we move to 4 and 8 clusters, the DCG-based tree gradually partitions the characters based on their colors. Note that the nuances within each color are not captured. For example, the yellow and orange characters remain in the same clusters. The equivalent hierarchical partitioning based on the Euclidean distance is much less visually intuitive (Fig 6(b)).

## Discussion

Clustering is a generic concept ubiquitous to data science, as it is easier to think about groups of data and representatives of those groups, rather than of the data themselves. Clustering however is difficult and there are no methods today that can be safely said to solve this problem. Exiting methods rely on different interpretation of the representation of the data points to be clustered, of the distance or similarity measures on those data, on the methods used to detect the manifolds on which those data lie, and even what defines clusters. In this paper, we focused on the distance measure and how it can be used to detect the geometry of the data points. We have proposed a method to derive a distance measure that captures this intrinsic geometry by scanning over its possible scales. The results of those scans are combined into a distance matrix between the data points; this distance matrix is shown to correspond to an ultrametric. We have compared this ultrametric measure to traditional distance measures on a series of toy problems, synthetic benchmarks, and real data sets and have demonstrated significant improvement.

The idea of relying on the geometry of the data points to cluster them is not new. ISOMAP [4] and spectral clustering methods [13] all define a local scale computed from the data point themselves, and filter the distances between the data points to emphasize the short distances that capture the local geometry. Diffusion maps techniques [6] further explore beyond that local geometry by defining time-dependent Markov chains on the data, with increasing values of the time (i.e. number of steps in the chain), to explore geometry. The approach we have proposed is dual to that approach. We still define Markov chains, but we fix their lengths, and scan instead the values of the scale defining local geometry. We do not claim that our approach is better than the concept of diffusion map: it is instead complementary and the two methods should in fact be combined. Indeed, our current implementation relies on the choice of the lengths of the Markov chains on the data, the parameter *t*. We have not found a systematic method for finding an optimal value for this parameter. It is also constrained for pragmatic reasons: the time complexity of our algorithm is linear in *t*; for large data sets, this can be a problem. We are currently exploring mechanisms in which both the value for the local scale of the data, our variable *T*, and the number of steps in the Markov chains, *t*, are varied.

There are other parameters in our method that require attention. The program requests as input an upper limit to the number of clusters in the data. For synthetic test cases, as those described in this paper, and for some real data sets, knowledge of the data makes it easy to define this parameter. There are however data sets for which this number is only assumed. The resulting matrix will be influenced by the choice made. In addition, for each temperature, we perform an eigen analysis of the Laplacian of the ensemble matrix derived from the different Markov chains built on the data. The first step of this analysis is to define the number of relevant eigenvalues. We rely on the concept of spectral gap and have implemented a pragmatic approach for detecting this gap. Much remains to be done however to make this procedure robust. This is in fact a known problem in spectral clustering method [55].

Our current implementation of the algorithm for computing the ultrametric matrix, DCG++, is slow, but manageable for medium size data sets. If *N* is the number of data points, computation at each temperature requires *NtP* operations (where *t* is the number of steps, and *P* the number of repeats) for the random walks, *kN*^2^ operations for computing the eigenvalues and eigenvectors of the ensemble matrix, where *k* is the number of iterations for the Lanczos algorithm, and *k*′*MKN* operations for the K-means algorithm, where *k*′ is the number of K-means iterations, *K* is the number of clusters considered, and *M* is the number of eigenvectors considered. Those calculations have been parallelized. This whole process needs to be repeated many times, at least twice the maximum number of clusters set as input. In practice, we observe an apparent *N*^3^ behavior, which is slow when *N* is large. A typical run however on a data set of 1000 points, with *t* = 1000, *P* = 10, and a request for up to 20 clusters takes 110 s wall time and 700 s CPU time on an Apple computer with an Intel i7 4GHz processor with 4 cores and 64 Gb of RAM, using the parallel features of DCG++. We are currently working on developing a better optimized algorithm.
